# Optimizing Real-Time Object Detection in a Multi-Neural Processing Unit System †

**DOI:** 10.3390/s25051376

**Published:** 2025-02-24

**Authors:** Sehyeon Oh, Yongin Kwon, Jemin Lee

**Affiliations:** 1 Department of Artificial Intelligence, University of Science and Technology, Daejeon 34113, Republic of Korea or osehn@etri.re.kr (S.O.); yongin.kwon@etri.re.kr (Y.K.); 2 Electronics and Telecommunications Research Institute, Daejeon 34129, Republic of Korea

**Keywords:** double buffering, queue-based processing, YOLOv3, neural processing unit, real-time object detection

## Abstract

Real-time object detection demands high throughput and low latency, necessitating the use of hardware accelerators. NPU is specialized hardware designed to accelerate the calculation of deep learning models, providing better energy efficiency and parallel processing performance than existing CPUs or GPUs. In particular, it plays an important role in reducing latency and improving processing speed in applications that require real-time processing. In this paper, we construct a real-time object detection system based on YOLOv3, utilizing Neubla’s Antara NPU, and propose two approaches for performance optimization. First, we ensure the continuity of NPU inference by allowing the CPU to process data in advance through double buffering. Second, in a multi-NPU environment, we distribute tasks among NPUs through queue-based processing and analyze the performance limits using Amdahl’s law. Experimental results demonstrate that compared to a CPU-only environment, applying the NPU in single buffering improved throughput by 2.13 times, double buffering by 3.35 times, and in a multi-NPU environment by 4.81 times. Latency decreased by 1.6 times in single and double buffering, and by 1.18 times in the multi-NPU environment. The accuracy remained consistent, with 31.4 mAP on the CPU and 31.8 mAP on the NPU.

## 1. Introduction

Recent advancements in artificial intelligence have increased demand for real-time video analysis and object detection applications [1,2]. However, as deep learning models become more complex, traditional CPU- or GPU-based systems struggle to meet real-time processing requirements [3]. To address these challenges, specialized hardware accelerators, such as Google’s TPU [4], NVIDIA’s Tensor Core [5], and Intel’s Nervana NNP [6], have been developed. These hardware accelerators are designed to maximize the efficiency of deep learning operations and contribute to improving computational performance based on their different architectural characteristics. The NPU has an architecture optimized for tensor operations and DNN calculations, and offers better parallel processing performance and energy efficiency than CPUs and GPUs. In particular, the NPU plays an important role in alleviating performance bottlenecks that occur in time-sensitive applications such as real-time object detection through low power consumption and high processing speed.

However, in real-time object detection systems, effectively utilizing the NPU requires cooperation with the CPU. Such a system follows a sequential process consisting of the preprocessing, inference, and postprocessing stages. The preprocessing retrieves and transforms the input data, after which the NPU performs inference, and finally, the postprocessing interprets and presents the results. However, data transmission delays, synchronization overhead, and inefficient resource utilization between the CPU and NPU can lead to performance bottlenecks. In particular, if each processor must wait for the other to complete its task before proceeding, latency increases, and system resources remain idle. This issue can reduce the overall system throughput and make it difficult to achieve real-time performance.

In this paper, to address these challenges, we propose a real-time object detection system based on YOLOv3 utilizing Neubla’s Antara NPU, with performance optimization achieved through a double buffering technique and a queue-based processing scheme for multi-NPU environments. The double buffering technique mitigates data processing bottlenecks by allowing the NPU to continue inference while the CPU processes data, alternating between two buffers. This enables parallel task execution between the CPU and NPU, maximizing system performance. The queue-based processing scheme uses input and output queues to distribute tasks evenly across NPUs in a multi-NPU environment.

The experimental results showed that, compared to a CPU-only system, the Antara NPU system with single buffering achieved approximately 2.13 times higher throughput, while the double-buffering system achieved about 3.35 times higher throughput. Latency in both single- and double-buffering systems was reduced by approximately 1.6 times compared to the CPU. In a multi-NPU environment, adding NPUs increased throughput by about 4.81 times compared to the CPU-only system; however, performance did not increase linearly due to CPU resource limitations. Additionally, inference using quantized models on the NPU maintained stable accuracy, with a slight increase from 31.4 to 31.8 mAP.

## 2. Related Work

### 2.1. Object Detection

Object detection models have evolved to balance accuracy and speed. These models are broadly classified into two-stage and one-stage detectors. Two-stage detectors, such as R-CNN, first generate candidate regions and then extract features to classify objects [7,8,9]. While this approach provides high accuracy, it involves multiple computational steps, resulting in relatively slower processing. In contrast, one-stage detectors, including YOLO and SSD, eliminate the candidate region generation step and directly predict object locations and classes in a single network pass. YOLO formulates object detection as a regression problem, enabling real-time inference [10]. SSD enhances small object detection by leveraging multi-scale feature maps [11]. Recently, models in the YOLO family and EfficientDet have shown gradual improvements in object detection compared to existing methods. YOLOv7 introduced a model re-parameterization technique to improve efficiency and accuracy at the same time [12]. YOLOv8 improved inference speed by applying an anchor-free object detection method. In addition, EfficientDet uses the Compound Scaling technique to adjust the depth, width, and input resolution of the model simultaneously, helping to strike a balance between lightness and performance [13] Recent research has focused on hardware acceleration techniques, optimizing computation using TensorRT, TPUs, and FPGAs. Neural processing units (NPUs) further enhance efficiency by optimizing parallel computation beyond traditional GPU-based processing. Our work explores the optimization of YOLOv3-based object detection on NPUs. By maximizing NPU parallel processing capabilities, we aim to improve FPS while maintaining accuracy.

### 2.2. Neural Processing Units

Research on hardware accelerators for deep learning computation optimization is actively progressing. NPUs are dedicated accelerators designed for deep learning, enabling high-speed tensor and matrix computations. While CPUs and GPUs have improved performance through parallel processing, the increasing computational demands necessitate specialized hardware. As a result, various companies are developing hardware and software stacks for deep learning optimization. Google provides the Coral TPU, which supports optimized inference using TensorFlow Lite and Edge TPU Runtime. Intel offers the Myriad X VPU along with the OpenVINO toolkit, enabling the conversion and optimization of various deep learning models. Neubla provides the Antara NPU and a dedicated software stack that supports optimized execution of ONNX models. Our work focuses on optimizing object detection models using the Antara NPU and applies optimization techniques to the software stack to maximize computational efficiency. Through this approach, we aim to enhance deep learning model performance and analyze the computational efficiency differences compared to GPU-based models.

## 3. Architecture and Execution Flow of Antara NPU

Neubla’s Antara NPU is optimized for real-time deep learning applications such as image classification, object detection, and super-resolution. It is designed to deliver high computational performance and efficient memory utilization. The Antara NPU features a 32 × 48 MAC array, supporting Int8/FP8 data types, and achieves 6 TOPS of computational power with 1.5 MB of on-chip SRAM to maximize bandwidth efficiency between computation and memory. Additionally, its 3000 MAC units provide fast and accurate inference performance. Antara NPU employs a design that maximizes the reuse of weights in convolutional operations, minimizing memory bandwidth while maintaining high computational intensity. This characteristic is particularly advantageous for computationally intensive applications such as real-time object detection. In addition, Antara NPU maintains a continuous data flow through partial sum-based operations and provides a simplified structure that is specialized for integer operations, thereby achieving high energy efficiency. This structure is optimized to deliver outstanding performance in real-time applications with less power consumption compared to hardware such as Google TPU or NVIDIA Tensor Core. The specifications of Neubla’s Antara NPU, compared with other NPUs, are summarized in Table 1.

**Figure 1:**
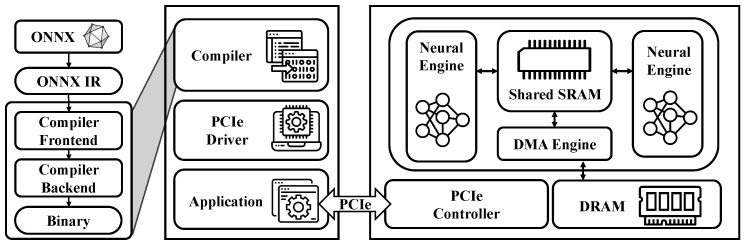
Neubla’s Antara NPU architecture and compiler.

As shown in Figure 1, the internal architecture of the Antara NPU consists of Neural Engines, shared SRAM, a DMA engine, and DRAM. Data are transferred from the host system to the DRAM on the Antara board via the PCIe interface, enabling the real-time processing of large-scale data. Additionally, the shared SRAM allows for efficient data exchange between the Neural Engines and the DMA engine, optimizing memory bandwidth efficiency. This architecture minimizes latency while providing the high throughput required for real-time applications, ensuring that the Neubla’s Antara NPU delivers the performance necessary for high-performance real-time deep learning applications.

The software stack includes a dedicated compiler that partitions the workload between the CPU and the NPU, enabling the execution of deep learning models on the Antara NPU. This compiler analyzes the model to divide it into parts that run on the CPU and the NPU, generating target binary files for the NPU execution. During this process, the CPU still handles preprocessing and postprocessing tasks, making efficient scheduling at the application level critical for coordinating tasks between the CPU and NPU. Our approach focuses on optimizing this scheduling to minimize idle time between CPU and NPU operations, thereby maximizing throughput. Further details on the pipeline optimization techniques are provided in the subsequent sections.

## 4. Best Practice for Efficient Object Detection on Antara NPU

### 4.1. Task Analysis

**Figure 2:**
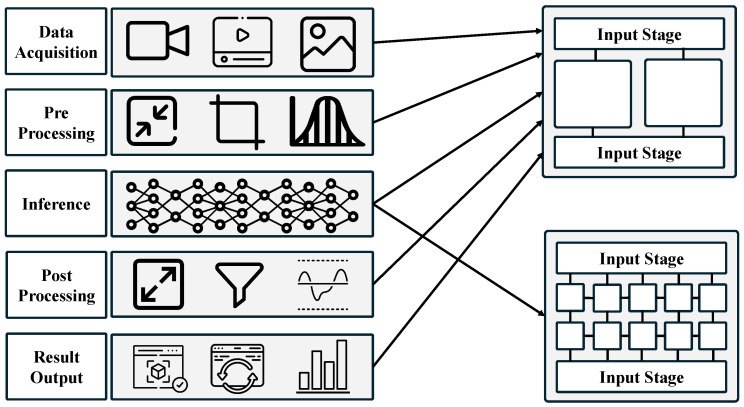
Workflow of object detection tasks, illustrating the stages from data acquisition to result output and their interactions with processing units.

Figure 2 is an analysis of the object detection system. In the data acquisition stage, data are collected from sources such as cameras, videos, and images. In the preprocessing stage, operations such as resizing, cropping, and normalization are performed to refine the data. In the inference stage, a trained model is used to analyze the preprocessed data and detect objects. In the postprocessing stage, techniques such as filtering and threshold adjustment are applied to enhance the detection results. In the result output stage, the postprocessed results are presented through visualization and updates to facilitate human interpretation. Among these stages, data acquisition, preprocessing, postprocessing, and result output are primarily processed on the CPU, while the inference stage can utilize both the CPU and NPU.

**Figure 3:**
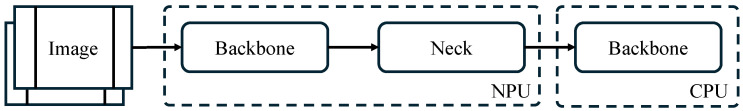
Object detection model design pattern with Backbone, Neck, and Head.

As shown in Figure 3, object detection models generally follow a design pattern consisting of a Backbone, Neck, and Head. The Backbone is responsible for extracting features from the input data and effectively processes features of various sizes and shapes using networks such as Darknet, ResNet, and EfficientNet. These operations require extensive parallel computation, making them well suited for processing on an NPU. The Neck processes and refines the features extracted by the Backbone, enabling the effective fusion of multi-scale information. This enhances the model’s ability to detect objects of varying sizes and positions while maintaining robust feature representation. Given its computational nature, the Neck also benefits from execution on an NPU. In contrast, the Head utilizes the features extracted by the Backbone to predict the locations and classes of objects in the final output stage, performing tasks such as bounding box regression and class prediction. Since this stage primarily involves computationally lighter tasks, it is more efficiently handled by a CPU. By designing the system to process the Backbone and Neck on the NPU and the Head on the CPU, the efficiency of hardware resource utilization can be maximized, thereby contributing to the overall optimization of object detection system performance.

### 4.2. Model Quantization and Compilation

The model was optimized and used in its compiled form through the Neubla Compiler after applying asymmetric quantization. During the quantization process, the moving average correction method was applied, and the model was converted to 8-bit precision. The Neubla Compiler provides various options to optimize execution. The Layer-wise (LW) option processes each layer by grouping it separately. The Exhaustive Group Search (EGS) option explores executable operations to find the optimal combination for grouping, efficiently bundling as many operations as possible while considering dependencies and memory usage. Additionally, the Single option allows a single TX Engine to compute the output Feature Map in 64-channel units, whereas the Half option splits the computation across two TX Engines, each handling 64-channel units. By applying this combination of options, the YOLOv3 model was compiled, and its execution speed is shown in Table 2.

### 4.3. Double Buffering and Queue-Based Processing

**Figure 4:**
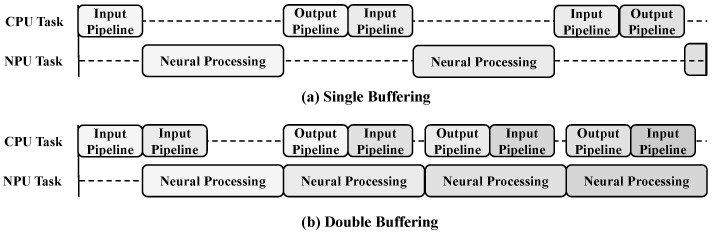
Single buffering with sequential operation and double buffering for parallel processing, where the timing differences between CPU and NPU tasks in each method are presented.

To optimize the performance of a real-time object detection system, the process is structured into input pipeline for data acquisition and preprocessing, neural processing for Backbone and Neck computations, and output pipeline for Head computation, postprocessing, and result output. In the typical processing flow, the CPU executes the input pipeline, then transfers the data to the NPU for neural processing, and finally, the CPU processes the results generated by the NPU through the output pipeline. In the initial implementation, a single buffering method was employed, where tasks between the NPU and CPU were executed sequentially. As shown in Figure 4a, the CPU completes the input pipeline before sending the data to the NPU, and after the NPU finishes neural processing, the CPU performs the output pipeline. However, in this approach, if the CPU does not prepare the next input data while the NPU is processing, the NPU remains idle, leading to a performance bottleneck. This reduces the overall system throughput and prevents optimal utilization of the NPU’s computational capabilities.

To address this performance bottleneck, a double-buffering technique was introduced to enable parallel processing between the CPU and NPU. In this approach, two buffers are used alternately, allowing the CPU to process input data in one buffer while the NPU performs neural processing in the other. This allows both the CPU and NPU to operate simultaneously, minimizing NPU idle time and significantly improving system processing speed. Particularly, in time-sensitive applications such as real-time object detection, this method reduces latency and contributes to performance optimization. As shown in Figure 4b, double buffering maximizes resource utilization and effectively increases throughput. Single buffering has the advantage of minimizing memory usage because only one buffer is allocated at a time. However, it has the disadvantage of limiting the overall throughput because the NPU is idle while the CPU is processing tasks. On the other hand, double buffering enables parallel processing, which can significantly reduce the idle time of the NPU and improve system performance. However, double buffering comes at the cost of increased memory usage, as two buffers must be allocated to handle CPU and NPU work alternately. This increase in memory usage is generally tolerable in high-performance systems, but can cause difficulties in environments with limited memory resources, such as embedded systems. Therefore, double buffering provides a trade-off between performance improvement and resource utilization, and this should be carefully considered to suit the requirements of the target application.

**Figure 5:**
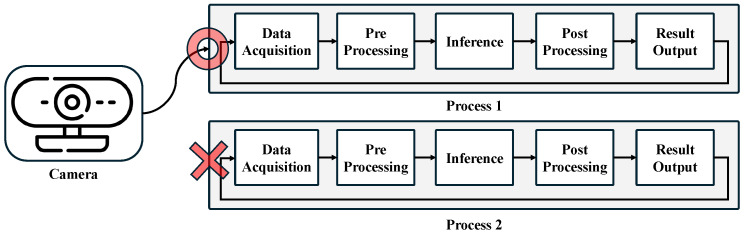
Issues of camera ownership across threads.

Queue-based processing was introduced to address the camera ownership issue in multi-NPU environments. In such environments, each NPU is required to process real-time video data. However, since a camera grants ownership to only a single entity, multiple NPUs cannot simultaneously access the camera’s data, leading to a resource contention problem. As shown in Figure 5, if one NPU occupies the camera, other NPUs are unable to utilize the same video data, thereby preventing efficient parallel processing. Due to this limitation, conventional systems face challenges in utilizing multiple NPUs with a single camera, often requiring a separate camera for each NPU, resulting in an inefficient hardware allocation strategy. Ultimately, this restriction reduces system scalability and limits the effective utilization of hardware resources.

**Figure 6:**
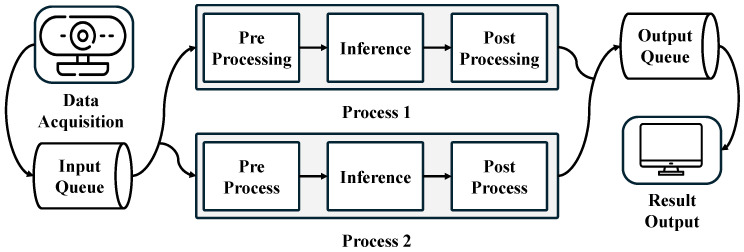
Data distribution through input and output queues in a multi-NPU system.

To achieve performance scaling in a multi-NPU environment, we implemented a queue-based processing strategy, as shown in Figure 6 The CPU acquires images from the camera in real-time and stores them in the input queue. Subsequently, the CPU handles preprocessing and postprocessing, while the NPU performs inference. These three stages are combined into a single task for each image until processing is complete. Each task retrieves data from the input queue, processes them, and stores the final output in the output queue, after which the CPU visualizes the results. This queue-based approach efficiently distributes the workload across the NPUs, maximizing the utilization of system resources. As each NPU processes data independently, increasing the number of NPUs can lead to an increase in system throughput. However, this performance gain becomes limited as the number of NPUs increases, due to constraints on system resources such as CPU, memory bandwidth, and I/O. Therefore, while the system can scale efficiently up to a certain point, it is important to recognize that performance may not improve proportionally with the addition of more NPUs [14].

## 5. Experiment

### 5.1. Environment Setup

To quantitatively evaluate the performance of a real-time object detection system and compare the performance differences across various hardware configurations, we designed experiments using an Intel Core i7-12700 CPU, an NVIDIA GeForce GTX 1060 6 GB GPU, and a Neubla Antara NPU. The experiments were conducted based on the YOLOv3 608×608 model, which was utilized in its UINT8 quantized version. Input data were provided in real time via a Logitech Brio 4K Pro camera, and all software was configured to run in a consistent environment to ensure the reliability of the experiments. The operating system used was Ubuntu 22.04, and inference on the CPU and GPU was performed using ONNX Runtime 1.12.1.

**Figure 7:**
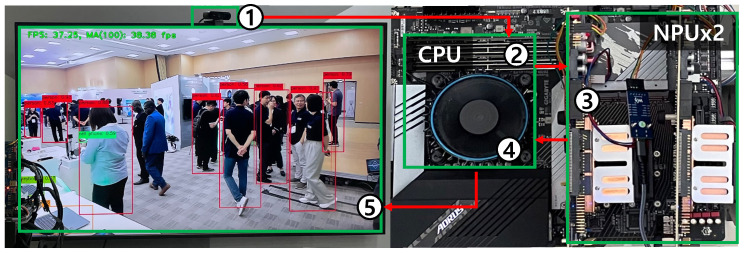
Five-stage execution flow: (1) data acquisition, (2) preprocessing, (3) inference, (4) postprocessing, and (5) real-time result output with object detection and performance metrics.

The overall execution flow is shown in Figure 7. This flow consists of five stages. ❶ Data acquisition, where real-time video data are collected from the camera. ❷ Preprocessing: the CPU performs preprocessing steps such as image resizing and data normalization. ❸ Inference, during which the preprocessed data are sent to the NPU or GPU for inference using the deep learning model. ❹ Postprocessing, in which the inference results are returned to the CPU, where tasks such as drawing bounding boxes around detected objects and organizing class information are performed. ❺ Result output, where the postprocessed results are displayed on the screen in real time, with detected objects indicated by bounding boxes and performance metrics such as Frames Per Second (FPS).

For performance evaluation, we measured the mean Average Precision (mAP) using the COCO 2017 validation dataset [15]. In addition, we assessed throughput (FPS) and latency for each hardware configuration to comprehensively analyze the performance of the real-time system. This allowed us to compare the inference performance across CPU, GPU, and NPU environments, providing a quantitative understanding of the performance differences between the hardware components.

### 5.2. Evaluation

Before evaluating the NPU system, the execution speed of the YOLOv3 model was measured across various hardware environments. To this end, the execution times on both the CPU and GPU were measured, and the results can be found in Table 3 and Table 4. In the GPU environment, the execution time of the FP32 model was measured, while the UINT8 model was excluded from the evaluation due to the lack of support for certain quantization operations. In contrast, both FP32 and UINT8 models were analyzed in the CPU environment. Performance comparison between CPU and GPU is a crucial factor in evaluating the level of computational optimization for different hardware. In particular, when executing a model using ONNX Runtime, unsupported operators on the GPU can be handled by the CPU. This distribution of computations affects execution speed, and even when utilizing GPU acceleration, performance may vary depending on the extent of CPU involvement. Therefore, it is essential to consider operator support when executing models and to establish an appropriate optimization strategy accordingly.

**Figure 8:**
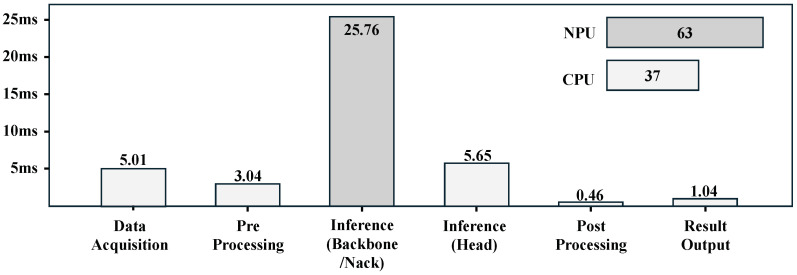
Stage-wise time analysis of object detection.

Furthermore, an analysis of the processing time for each stage of the object detection system was conducted to identify potential bottlenecks. The object detection system follows a sequential process comprising data acquisition, preprocessing, Backbone and Neck operations, Head operations, postprocessing, and result output. The measured execution times for each stage are shown in Figure 8. The results indicate that data acquisition takes 5.01 ms, preprocessing takes 3.04 ms, Backbone and Neck operations require 25.76 ms, Head operations take 5.65 ms, postprocessing requires 0.46 ms, and result output takes 1.04 ms. In this study, video acquisition and preprocessing are performed on the CPU, while Backbone and Neck operations are executed on the NPU. Subsequently, the Head operations and result output stages are processed again on the CPU. The analysis reveals that the proportion of total computation time allocated to the CPU and NPU is 37% and 63%, respectively.

Finally, a comprehensive analysis was conducted to evaluate the performance differences across various hardware configurations. To this end, throughput (FPS), latency, and accuracy were compared, and the detailed results are summarized in Table 5. In the case of the CPU-only (Baseline) in integer operation mode, a throughput of 11.14 FPS was recorded, with a latency of 99.73 ms and an accuracy of 31.4 mAP. When using the NPU, the performance in the single-buffering (SB) configuration shows that the CPU preprocesses data in 8.05 ms, the NPU performing inference takes 25.76 ms, and the CPU postprocesses the result in 7.15 ms, resulting in a total processing time of 40.96 ms. Based on this, the calculated FPS is 24.41, which represents approximately 2.13 times the performance improvement compared to using the CPU alone. In the case of double buffering (DB), since each stage is processed in parallel, the overall processing time is determined by the longest stage, which is the inference time of 25.76 ms. Therefore, the calculated FPS is 38.79, which is close to the actual measured value of 38.38 FPS. This indicates that double buffering improves throughput by approximately 3.35 times compared to CPU-only usage, and by 1.58 times compared to single buffering. In terms of latency, the measured latency for double buffering is 37.77 ms, which shows little difference from the 38.34ms observed in single buffering. This demonstrates that double buffering increases throughput without significantly increasing latency.

In a multi-NPU environment, we conducted experiments using two NPUs and optimized data distribution through a queue-based processing method. As a result, the throughput increased to 55.04 FPS. However, the improvement was sublinear, indicating that the CPU’s preprocessing and postprocessing tasks became bottlenecks. Analysis showed that approximately 63% of the total workload is parallelizable, primarily corresponding to the inference stage. Applying Amdahl’s law, which relates the proportion of parallelizable tasks to overall performance improvement, we calculated the theoretical speedup using the formula $S=\frac{1}{\left(1-P\right)+\frac{P}{N}}$, where P=0.63 and N=2. Substituting these values, we obtain a speedup of 1.46. Therefore, the expected FPS is 56.03, which closely matches the actual measured value of 55.04 FPS. This demonstrates that even with multiple NPUs, performance improvement is limited by the non-parallelizable portions of the workload and CPU resource constraints.

In terms of accuracy, the differences between hardware were minimal. CPU recorded 31.4 mAP, while the NPU showed a slightly higher value at 31.8 mAP. This small variation may be attributed to differences in how quantized models are executed across hardware [16]. Notably, the NPU maintained model accuracy while improving processing speed and reducing latency.

The results of the experiment, including the operation of FP32 mode on the GPU, are as follows. In the CPU-only FP32 mode, the throughput decreased from 11.14 FPS to 5.37 FPS due to the increased calculation load, resulting in a decrease in performance. However, the latency-to-throughput ratio increased by 1.006 times, indicating that the balance of the latency-to-throughput ratio was almost maintained despite the increased calculation load. In the case of Jetson AGX Orin, the throughput was 5.68 FPS, and the latency increased by 1.486 times compared to the throughput. In other words, the latency-to-throughput ratio increased, indicating that latency efficiency was lower than when using only the CPU. For the GTX 1060 6 GB GPU, the throughput was 14.02 FPS, and the latency-to-throughput ratio increased by 1.114 times. This means that latency efficiency is relatively better than that of the Jetson AGX Orin, but latency increases compared to using only the CPU. This result suggests that while GPUs excel at processing large batch sizes, their higher latency may make them less suitable for real-time applications, where NPUs offer better responsiveness.

## 6. Discussion and Limitations

This study proposes two optimization techniques, double buffering and queue-based processing, for NPU-based real-time object detection systems, and verifies the effects of throughput and latency improvement through these techniques. However, there are some limitations to this study. First, this study conducted experiments on the YOLOv3 model, which may limit the generalizability of the research results. The core of this study is to present an optimization technique that is not dependent on a specific model, and it is believed that the proposed mechanism can be applied to the latest model if the appropriate compiler and hardware support are secured. The reason for choosing YOLOv3 is that, due to the nature of this study, which is based on custom compilers and dedicated hardware environments of companies and institutions, compiler modifications to support the latest model would exceed the scope of this study.

In addition, the hardware configuration used in this study is based on a prototype NPU, so there are constraints and cost issues that must be considered in actual industrial deployment, such as the PCIe slot-based host system, physical installation space, power supply method, and PCIe communication compatibility. However, the proposed NPU has a simple structure designed for inference only, so it is expected to be miniaturized and low-power in the future.

In addition, in a multi-NPU configuration, the tasks performed by the CPU, such as preprocessing and postprocessing, have a significant impact on overall system performance. As the number of NPU increases, the parallel processing benefits of the inference stage increase, but if the CPU performs preprocessing and postprocessing tasks with a single or limited thread, a bottleneck occurs, limiting the linear improvement in overall throughput. This phenomenon is consistent with Amdahl’s law, and this study also confirmed the performance limitations caused by the CPU bottleneck in a multi-NPU environment. Future research will need to mitigate this limitation by parallelizing preprocessing and postprocessing or introducing additional hardware accelerators.

## 7. Conclusions

In this paper, we proposed two approaches to optimize the performance of a real-time object detection system utilizing Neubla’s Antara NPU. First, by employing double buffering, the CPU processed data in advance, ensuring continuity in NPU inference. Second, in a multi-NPU environment, a queue-based processing scheme was introduced to distribute tasks across NPUs, and the performance limits were analyzed using Amdahl’s law [17]. Experimental results showed that, compared to the CPU-only system, the throughput increased by 2.13 times with single buffering, 3.35 times with double buffering, and 4.81 times in the multi-NPU environment. Latency was reduced by 1.6 times with single and double buffering and by 1.18 times in the multi-NPU setup. Accuracy remained consistent, with 31.4 mAP on the CPU and 31.8 mAP on the NPU. This study shows that a real-time object detection system using NPU can provide high efficiency and performance, and emphasizes the importance of task distribution and resource utilization in a multi-NPU environment. Future research will evaluate the proposed technique’s universality and scalability by applying it to other accelerator architectures, such as Google TPU or NVIDIA GPUs with Tensor Cores. This research has contributed to the optimization of NPU-based object detection systems and will serve as a basis for the advancement of real-time object detection technology. 

## Figures and Tables

**Table 1:** Comparison of hardware specifications: Neubla’s Antara, Google’s Coral EdgeTPU, and Hailo-8.

Feature	Antara	Coral EdgeTPU	Hailo-8
Performance	6 TOPS	4 TOPS	26 TOPS
Embedded SRAM	1.5 MB	8 MB	N/A
# of MACs	3 K (1.5 K × 2)	N/A	N/A
Data Type	Int-8	Int-8	Int-8
TOPS/W	4	2	2.9

**Table 2:** Comparison of model performance by compilation options. LW refers to Layer-wise execution, and EGS stands for Exhaustive Group Search.

Configuration	LW, Single	LW, Half	EGS, Single	EGS, Half
Execution Time (ms)	56.80 ± 0.48	48.13 ± 0.51	32.87 ± 0.47	26.06 ± 0.50

**Table 3:** Inference speed comparison of different GPUs using an FP32 model.

Config.	Jetson AGX Orin 64 GB	GTX 1060 6 GB	RTX 2080 TI 11 GB	RTX 3080 10 GB
FP32 (ms)	99.03 ± 0.33	56.13 ± 0.43	15.19 ± 0.06	12.11 ± 0.03

**Table 4:** Inference speed comparison of different CPUs using FP32 and UINT8 models.

Config.	ARM Cortex-A78AE	i7-12700	i9-12900	i7-9700K
FP32 (ms)	6009.34 ± 4.69	171.13 ± 6.56	177.59 ± 0.29	1014.25 ± 1.61
UINT8 (ms)	1688.57 ± 2.84	73.21 ± 4.25	89.69 ± 0.25	910.94 ± 10.00

**Table 5:** Performance comparison across different hardware configurations. Configurations marked with * use double buffering. Configurations without * use single buffering. Configurations marked with † represent FP32 operations, while others represent UINT8 operations.

HW Config.	Throughput (FPS)	Latency (ms)	Accuracy (mAP)
Intel i7-12700 CPU (Baseline)	11.14	99.73	31.4
AntaraNPU	24.41	38.34	31.8
AntaraNPU *	38.38	37.77	31.8
AntaraNPU×2 *	55.04	81.88	31.8
$\mathrm{Intel}\;i7\text{-}12700\;{\mathrm{CPU}}^{\;†}$	5.37	187.43	31.4
$\mathrm{Jetson}\;\mathrm{AGX}\;\mathrm{Orin}\;{\mathrm{GPU}}^{\;†}$	5.68	261.61	31.4
$\mathrm{GTX}\;1060\;6\;\mathrm{GB}\;{\mathrm{GPU}}^{\;†}$	14.02	79.48	31.4

## Data Availability

Data are contained within the article.
